# Reciprocal REG*γ*-Nrf2 Regulation Promotes Long Period ROS Scavenging in Oxidative Stress-Induced Cell Aging

**DOI:** 10.1155/2023/4743885

**Published:** 2023-01-10

**Authors:** Solomon Kibreab, Zimeng Wang, Xiangzhan Zhu, Yuping Ren, Yiming Jing, Xiaotao Li, Lei Li, Bianhong Zhang

**Affiliations:** Shanghai Key Laboratory of Regulatory Biology, Institute of Biomedical Sciences, School of Life Sciences, East China Normal University, 500 Dongchuan Road, Shanghai 200241, China

## Abstract

Increased accumulation of reactive oxygen species (ROS) and decline of adaptive response of antioxidants to oxidative stimuli has been implicated in the aging process. Nuclear factor erythroid 2-related factor 2 (Nrf2) activation is a core event in attenuating oxidative stress-associated aging. The activity is modulated by a more complex regulatory network. In this study, we demonstrate the proteasome activator REG*γ* function as a new regulator of Nrf2 activity upon oxidative stress in cell aging model induced by hydrogen peroxide (H_2_O_2_). REG*γ* deficiency promotes cell senescence in primary MEF cells after H_2_O_2_ treatment. Accordingly, ROS scavenging is accelerated in WT cells but blunted in REG*γ* lacking cells during 12-hour recovery from a 1-hour H_2_O_2_ treatment, indicating long-lasting antioxidant buffering capacity of REG*γ*. Mechanistically, through GSK-3*β* inhibition, REG*γ* enhances the nuclear distribution and transcriptional activity of Nrf2, which is surveyed by induction of phase II enzymes including Ho1 and Nqo1. Meanwhile, Nrf2 mediates the transcriptional activation of REG*γ* upon H_2_O_2_ stimulation. More interestingly, short-term exposure to H_2_O_2_ leads to transiently upregulation and gradually descent of REG*γ* transcription, however sustained higher REG*γ* protein level even in the absence of H_2_O_2_ for 24 hours. Thus, our results establish a positive feedback loop between REG*γ* and Nrf2 and a new layer of adaptive response after oxidative stimulation that is the REG*γ*-GSK-3*β*-Nrf2 pathway.

## 1. Introduction

Aerobic creatures are constantly exposed to oxidants, which may originate from internal sources, such as mitochondrial dysfunction or from external sources, such as exposure to hydrogen peroxide (H_2_O_2_) or other environmental pollutants [1, 2]. Oxidative stress in the cell is caused by an equilibrium disruption between formation of free radicals and their removal by enzymatic and nonenzymatic antioxidant molecules, which detoxify harmful effects and are primary lines of defense and critical for maintaining various cell functions [3–6]. The cumulative and increasing oxidative damage to cells has been linked to a variety of age-related pathologies [7, 8]. In addition, the decline of the repair systems, including the proteasomal degradation of damaged proteins and the adaptive response to oxidative stress is also associated with chronic oxidative state in aging. [9, 10]

Nuclear factor erythroid 2-related factor 2 (Nrf2), a major sensor of oxidative stress in the cell [11, 12], belongs to the basic leucine zipper transcription factor family featuring a cap'n'collar motif [13, 14]. Numerous studies have provided clear evidence that Nrf2 is a crucial molecule in the regulation of basal and induced expression of phase II genes through regulation of antioxidant response elements (AREs), also known as electrophile response element (EpRE) [15, 16]. Depending on the cellular redox balance, the Nrf2-ARE signaling system is subjected to multiple layers of regulation [17]. The main regulation is provided by Kelch-like-ECH-associated protein 1 (Keap1), a cullin-3- (Cul3-) based E3 ubiquitin ligase substrate adaptor, allowing Nrf2 to be ubiquitylated by Cul3/Rbx1 [18]. Under normal homeostatic situations, Nrf2 is sequestered by forming a complex with numerous cytoplasmic proteins, including Keap1 and targeted to proteasomal degradation [19], resulting in a low baseline level of Nrf2. In addition, *β*-TrCP, another E3 ligase adaptor also facilitates Nrf2 ubiquitination and degradation through the Skp1-Cul1-Rbx1/Roc1 core E3 complex [20]. Under the conditions of oxidative stress, Nrf2-Keap1 interaction is disrupted [21, 22], which leads Nrf2 translocation to the nucleus to increase the expression of protective genes and antioxidant enzymes including the phase II detoxifying enzymes heme oxygenase-1 (Ho1) and NAD(P)H quinone oxidoreductase 1 (Nqo1) [1, 23]. Posttranslational modification such as phosphorylation and sumoylation is also another regulatory layer of Nrf2 intracellular distribution, activity, and stability [24].

Glycogen synthase kinase-3*β* (GSK-3*β*) is a serine/threonine kinase and originally reported as a key enzyme of glucose homeostasis through regulation of the rate of glycogen synthesis. It has subsequently been found to influence most cellular processes, including growth, differentiation and death, as part of its role in modulating response to hormonal, nutritional, and cellular stress stimuli [25, 26]. Moreover, an increasing body of literature has indicated GSK-3*β* as a negative regulator of Nrf2. In the delayed/late response to oxidative stress, the Tyr216 of GSK-3*β* site is phosphorylated, and the activated GSK-3*β* acts upstream and phosphorylates Fyn kinase, which in turn translocates into nucleus and phosphorylates tyrosine 568 of Nrf2, leading to the nuclear export of Nrf2 and its turn over by proteasome [27]. Phosphorylation of Nrf2 Neh6 domain by GSK-3*β* also facilitates its interaction with *β*-TrCP and subsequent degradation. In contrary, chemicals or short interfering RNA mediated inhibition of GSK-3*β* results in nuclear accumulation of Nrf2 and transcriptional activation of the Nrf2 downstream gene *Nqo1* and *Ho1* [28, 29]. Nevertheless, after transient stress stimulation, how Nrf2 activation is maintained for a prolonged period remains largely unknown.

REG*γ* (also known as PA28*γ*, PSME3, or Ki antigen) belongs to the REG or 11S family of proteasome activators “caps” that have been shown to bind and activate the 20S core proteasome [30, 31]. It has been found to stimulate the degradation of a number of critical cellular regulatory proteins without the use of ubiquitin or ATP, including SRC-3, p21, p16, p19, PKAc*α*, CK1*δ*, SirT1, I*κ*B*ε*, GSK-3*β*, and Smad7 [32–35]. Moreover, REG*γ* facilitates the turnover of tumor suppressor p53 by facilitating MDM2-mediated p53 ubiquitination [36] and p53 cellular distribution regulation [37]. Furthermore, REG*γ* plays roles in the control of a variety of physiological and pathological processes, including cancer progression [38–40], energy metabolism [41, 42], bacterial infection [43], innate immunity, and inflammatory diseases [44, 45]. Previous work shows REG*γ* mediates regulation of aging through CK1-Mdm2-p53 pathway [46]. REG*γ* deficiency causes premature aging phenotypes. As part of the proteasomal system, REG*γ* involves in removing oxidatively damaged proteins under acute oxidative stress of mild or severe stimulation with H_2_O_2_ [47]. However, roles of REG*γ* in adaptive response during recovering from oxidative stress remain to be explored.

In this study, we found that REG*γ* deficiency accelerates cell senescence in H_2_O_2_-induced cell aging model. H_2_O_2_ activated Nrf2 to enhance REG*γ* expression, which maintained the nuclear location and activity of Nrf2 via inhibition of GSK-3*β*, resulting in decreased accumulation of reactive oxygen species (ROS) during 12-hour recovery from a 1-hour H_2_O_2_ treatment. Taken together, our results demonstrate a long-lasting proantioxidants function of REG*γ* in recovery from oxidative stress.

## 2. Materials and Methods

### 2.1. Animals

*REGγ^+/+^* and *REGγ^−/−^* mice were kindly provided by Dr. John Monaco at the University of Cincinnati College of Medicine [45]. Animal procedure was carried out in accordance with the American Association for the Accreditation of Laboratory Animal Care International's guidelines and was approved by the school's Animal Center.

### 2.2. Cell Culture and Reagent

HEK 293 cells were originally purchased from the ATCC (Manassas, USA). *REGγ^+/+^* and *REGγ^−/−^* MEF (murine embryonic fibroblast) were previously generated [48]. 293 or MEF cells were cultured with Dulbecco's modified Eagle's medium (DMEM) (Invitrogen), containing 10% fetal bovine serum (FBS) (Invitrogen) and 100 *μ*g/ml penicillin/streptomycin. All cells were cultured in a 37°C incubator with 5% CO_2_.

### 2.3. Primary Fibroblast Cultures from Mouse

The skin from mice were cut into small pieces approximately 2-3 cm and dissociated with dispase II rotated at 4°C overnight. The next day, the epidermis was peeled off and the dermis was cut into small pieces (1 mm) and transferred into the culture plate (6-wells or 3.5 cm dish). Then the cells were incubated at 37°C in 5% CO_2_, in DMEM (Invitrogen) supplemented with 10% FBS, and the medium was changed every day for 3-4 days.

### 2.4. Real-Time Quantitative RT-PCR (RT-qPCR) and Gel-Based PCR

Total RNA was isolated from cells using TRIzol reagent (Invitrogen). DNase treated RNA was reverse-transcribed with a reverse transcription kit (Vazyme, China). PCR products were analyzed using 7500 Fast Real-Time PCR System (Applied Biosystems, USA), and relative transcript abundance was normalized to that of 18S mRNA. The oligonucleotide primers for PCR are as follows. *18S*: Forward 5′ GGACACGGACAGGATTGACA-3′, Reverse, 5′-GACATCTAAGGGCATCACAG-3′; *Ho1:* Forward 5′-GCCGAGAATGCTGAGTTCATG-3′, Reverse, 5′ TGGTACAAGGAAGCCATCACC-3′; *Nqo1:* Forward 5′-CGCCTGAGCCCAGATATTGT-3′, Reverse 5′-GCACTCTCTCAAACCAGCCT-3′.

### 2.5. Western Blot Analysis

Whole cell proteins were extracted using radioimmunoprecipitation assay (RIPA) lysate (Beyotime, Shanghai, China). After the protein was separated by sodium dodecyl sulphate-polyacrylamide gel electrophoresis (SDS-PAGE), the target protein was transferred to the nitrocellulose filter (NC) membranes. After 5% of the skim milk powder was blocked for 2 h, it was incubated with a specific primary antibody at 4°C overnight. The next day, after washing with Tris-Buffered Saline-Tween (TBST), fluorescent-labeled (Jackson ImmunoResearch) secondary antibodies were added and incubated for 1 hour on a shaker. Then, bands were detected and visualized by a LI-COR Odyssey Infrared Imaging System. Specific primary antibody Nrf2, Rabbit, 1 : 1000 (Abcam); Ho1 and Nqo1, Rabbit, 1 : 1000 (Abcam); anti-*β*-actin-mouse 1 : 1000 (Sigma).

### 2.6. Senescence-Associated *β*-Galactosidase Staining Assay

To measure cellular senescence, cells were stained with the SA *β*-Gal staining kit (Beyotime Institute of Biotechnology, Nanjing, China) according to the manufacturer's instructions. Images containing >200 cells were taken using a bright-field microscopy and total, and blue-colored cells were counted. The percentage of SA *β*-Gal positive cells was represented by the ratio between the number of blue-colored cells and the number of total cells from at least three independent experiments.

### 2.7. Detection of Intracellular ROS

The intracellular levels of ROS were measured by loading the 293 or MEF cells with the fluoroprobe H2-dichlorofluorescein diacetate (H2-DCF-DA) (Invitrogen). Dichlorofluorescein diacetate (DCF-DA) taken up by cells, is cleaved to DCF by intracellular esterases, and is oxidized in the presence of ROS to DCF. The H2-DCF-DA probe was freshly reconstituted in DMSO before loading. In brief, cells were cultured 5 × 10^4^ cells per well of 12-well plate in 1 ml of complete medium for 24 h. For measurement of the effect of H_2_O_2_ treatment on ROS generation, the wells were washed with PBS after H_2_O_2_ treatment, loaded for 30 min at 37°C with 1 *μ*M DCF-DA in medium. Then, the DCF-DA containing medium was removed, cells were washed with PBS and maintained in PBS while fluorescence intensity was read by fluorescence-activated cell sorting (FACS). The fluorescence intensity was analyzed and quantified using FLOW JO (Tree Star). The results are representative of 3 independent cultures.

### 2.8. Oxidative Stress Induction

To induce oxidative stress, a stock solution of H_2_O_2_ (30%, Sigma, USA) was added directly to the culture medium to a final concentration of 50 *μ*M.

### 2.9. REG*γ* Luciferase Reporter Constructs

PCR was used to amplify DNA fragments containing REG*γ* genomic sequences from 293 T cell genomic DNA, and primers were derived from human genomic REG*γ* and ligated into the kpn1/xhol sites of the promoter less pGL4-Basic (Promega, Madison, USA) vector and was named as pGL4-REG*γ*-luc.

### 2.10. Luciferase Assay

After transfection, cells were collected and washed three times with cold PBS, followed by lysis in a cell lysis buffer (Promega, Madison, WI, USA). After one freezing and thawing cycle, whole-cell lysates were centrifuged in a cold room (4°C) at 12,000 rpm for 15 min, and the supernatant obtained was collected in a fresh tube. Next, 20 *μ*l supernatant was added to equal amounts of luciferase assay substrate, and luminescence was detected as relative light units by using the LUMIstar OPTIMA reader (BMG Labtech, Offenburg, Germany). Data were normalized with beta actin and obtained from three independent experiments. Fold change in values is represented as a mean of three experiments.

### 2.11. Immunofluorescence

The cells were fixed with 4% paraformaldehyde, and the goat serum was added drop wise for 1 h. The diluted antibody Nrf2 (1 : 500, Abcam, Cambridge, MA, USA) was added drop wise and placed in a refrigerator at 4°C overnight. The second antibody was added to the next day in the dark, and after 1 h of incubation, 4′,6-diamidino-2-phenylindole (DAPI) was stained and incubated in the dark. After 15 minutes, the images were taken by Leica laser confocal microscope.

### 2.12. Statistics

All data in the experimental analyses were generated using GraphPad Prism6 software. Significance among different groups was determined using two-tailed unpaired *t*-tests. All values were expressed as mean ± SD. Values of *p* < 0.05 were considered statistically different.

## 3. Results

### 3.1. REG*γ* Deficiency Accelerates Cell Senescence Induced by H_2_O_2_

**Figure 1:**
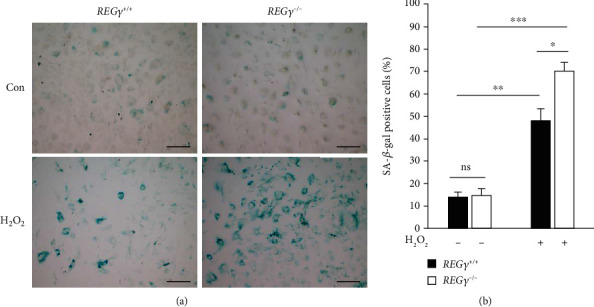
REG*γ* deficiency accelerates cell senescence induced by H_2_O_2_. (a) WT and REG*γ* knockout primary MEF cells were treated with 50 *μ*M H_2_O_2_ for 30 min and then stained with *β*-galactosidase staining kit 4 days later. Images were taken using a bright-field microscopy. Scale bars, 200 *μ*M. (b) Quantitative analysis of positive *β*-galactosidase-stained cells in REG*γ*^+/+^ and REG*γ*^−/−^ cells during cellular senescence. At least 600 cells from three independent experiments in each group were counted for quantification. Error bars represent ± SD. ^∗^*p* < 0.05, ^∗∗^*p* < 0.01, ^∗∗∗^*p* < 0.001, ns: nonsignificant, Con: control.

Previous studies have focused on immediate effect of REG*γ* upon stimulation with H_2_O_2_, such as degrading oxidatively damaged proteins [47]. In order to clarify the long-term function of REG*γ* during recovering from acute oxidative stress, we applied a cell senescence model induced by H_2_O_2_ treatment. SA-*β*-galactosidase activity assay was employed to monitor the cell aging. The results showed that SA-*β*-gal-positive cells were markedly increased in both WT and REG*γ* deficient primary MEF cells treated with H_2_O_2_ compared to nontreated cells (Figures 1(a) and 1(b)). More importantly, the percentage of SA-*β*-gal positive staining cells was more pronounced in REG*γ* lacking cells compared to those REG*γ* normal cells at day 4 post 30 minutes treatment with H_2_O_2_, which was in agreement with previous work that REG*γ* KO mice developed phenotype characteristic of premature aging [46]. But, there was no big difference in nontreated control parts (Figure 1(b)). These results manifest the protective function of REG*γ* in the process of cell aging.

### 3.2. REG*γ* Accelerates ROS Scavenging during Recovery from Short Exposure of H_2_O_2_

**Figure 2:**
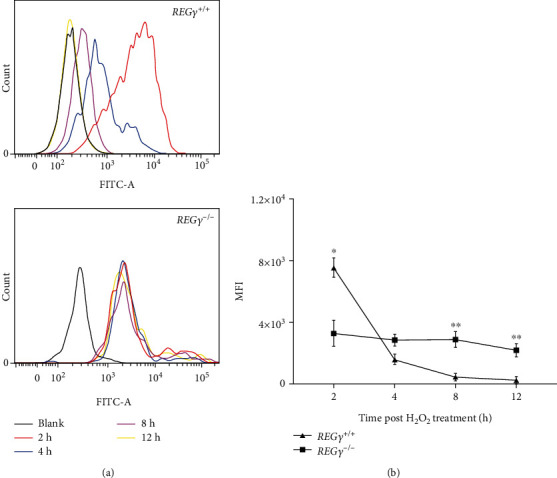
REG*γ* accelerates ROS scavenging after H_2_O_2_ treatment. (a) REG*γ* wild type and knockout MEF cells were stimulated with 50 *μ*M H_2_O_2_ for 30 min, then H_2_O_2_ was replaced by fresh medium and the cells were allowed to adapt for indicated time. DCFH-DA ROS fluorescence probe was added and incubated for 20 min, and then mean fluorescence intensity (MFI) was measured by flow cytometry. (b) Experiments were performed in triplicate. The changes of MFI values with the recovery time after oxidative stress were statistically analyzed. Error bars represent ± SD. ^∗^*p* < 0.05, ^∗∗^*p* < 0.01.

The link between the accumulation of reactive oxygen species (ROS) and cellular senescence has been well established [49, 50]. Therefore, the intracellular ROS level was immediately measured in REG*γ* WT and KO MEF cells after exposure in H_2_O_2_ for 2 hours. Surprisingly, we observed significantly higher ROS content in WT cells than REG*γ* knockout cells (Figures S1a and S1b) and the possible explanation will be discussed in the discussion part. We speculated that REG*γ* may have a long-lasting antioxidant effect, so we exposed cells to oxidative stress for a short period of time with H_2_O_2_ and continued routing culturing for up to 24 hours after H_2_O_2_ removal. The dynamic ROS level at different time point of recovery was tested. Interestingly, the results showed that the ROS level was gradually decreasing in REG*γ* WT MEF cells, however kept unaltered in REG*γ* deficient cells (Figure 2(a)). Starting from around 4-hour recovery, the ROS accumulation in REG*γ* containing cells was evidently less than in REG*γ* lacking cells (Figures 2(a) and 2(b)), which was consistent with the cellular senescence phenotype. Taken together, these data declare that REG*γ* expedites ROS scavenging and maintains it at a lower concentration during recovery from oxidative stress, suggesting its long-term antioxidant and antiaging capacity.

### 3.3. REG*γ* Promotes the Nuclear Retention and Activity of Nrf2 in Long-Term Recovery from Oxidative Stress

**Figure 3:**
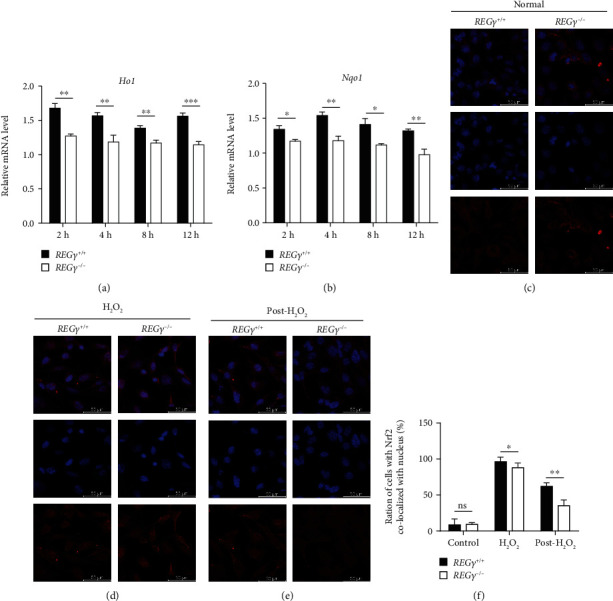
REG*γ* enhances Nrf2 nuclear retention after oxidative stress. (a, b) REG*γ* wild type and knockout primary MEF cells were subjected to qRT-PCR analysis to detect the expression of the downstream target genes of *Ho1* and *Nqo1* after removal of H_2_O_2_ for indicated time. (c) Immunofluorescence staining was performed using nonstressed control primary mouse fibroblast cells from REG*γ*-WT and KO mice. Red: Nrf2. Blue: DAPI. The images were taken using Leica laser confocal microscope. Original magnification, 40x. Scale bars, 50 *μ*M. (d) After exposure with 50 *μ*M H_2_O_2_ for 30 minutes, REG*γ* wild type and knockout primary MEF cells were immediately collected for immunofluorescence staining. Red: Nrf2. Blue: DAPI. Scale bars, 50 *μ*M. (e) After 30 min of 50 *μ*M H2O2 stimulation, fresh medium was replaced for further culture for 8 hours. Cells were immunostained with anti-Nrf2 antibody (red) and the nuclei were counterstained with DAPI (blue), and then the nuclear translocation of Nrf2 was observed by confocal laser-scanning microscope. Scale bars, 50 *μ*M. (f) 5 fields of view (>300 cells) were randomly selected from each group and the numbers of Nrf2-positive nuclear staining cells were counted for quantification by ImageJ software. The corresponding statistical analyses of the cells with Nrf2 nuclear localization (%) were presented as means ± SD. For a–f, at least three independent experiments were performed with similar results. ^∗^*p* < 0.05, ^∗∗^*p* < 0.01, ^∗∗∗^*p* < 0.001, ns: not significant.

In searching for the potential mechanisms of ROS eliminating by REG*γ*, we first speculated that REG*γ* might regulate the activity of Nrf2, because Nrf2 is a critical redox sensor and activates the transcription of a number of antioxidant genes that is known for combating ROS [51]. As expected, we observed a notably higher expression of Nrf2 target genes, including *Ho1* and *Nqo1* after 2 hours of recovery from H_2_O_2_-induced WT cell conditions in compare with the REG*γ*^−/−^ cell conditions (Figures 3(a) and 3(b)). Given proteasome activator function of REG*γ*, we wondered whether REG*γ* influences Nrf2 stability. To answer this question, MEF WT/KO cells were treated with the protein synthesis inhibitor cycloheximide (CHX). Results showed that the Nrf2 protein level decreased faster in REG*γ* KO cells compared to WT cells, suggesting that Nrf2 is not a direct substrate of REG*γ*-20S proteasome (Figure S2a and b). Next, immunofluorescence staining was executed to monitor the nuclear location of Nrf2 in primary mouse fibroblast cells from REG*γ*-deficient and REG*γ* wild type mice. There was no significant difference among the nonstressed control group (Figures 3(c) and 3(f)). However, H_2_O_2_ induced more visible nuclear localization of Nrf2 in REG*γ* containing cells than REG*γ* lacking cells, especially in condition that H_2_O_2_ had been removed for 8 hours (Figures 3(d)–3(f)). In summary, we draw the conclusion that REG*γ* regulates the Nrf2 activity by dominating its nuclear location.

### 3.4. REG*γ* Maintains the Activity of Nrf2 after Oxidative Stress via GSK-3*β*

**Figure 4:**
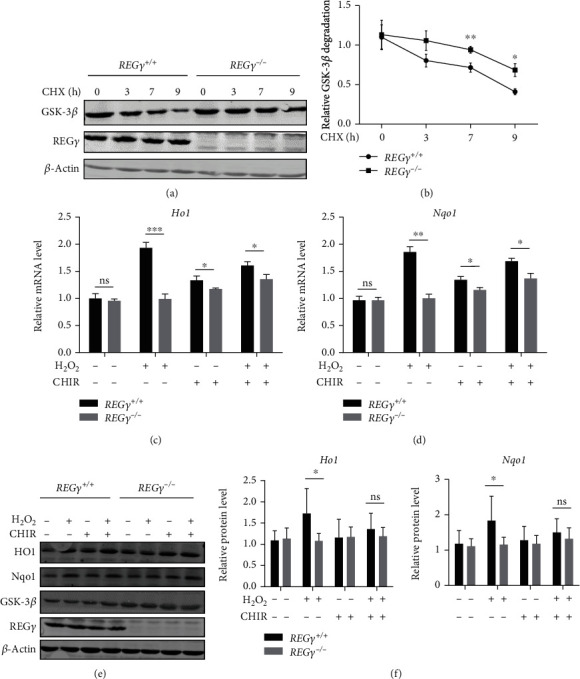
REG*γ* enhances oxidative stress-triggered Nrf2 activity via GSK-3*β*. (a) 100 *μ*g/mL cycloheximide was added to REG*γ* WT or KO MEF cells, and then the cells were harvested at indicated time. The stability of GSK-3*β* was detected by western blot. (b) Experiments in (a) were repeated for six times and GSK-3*β* degradation was analyzed quantitively by ImageJ software. The data presented as mean values ± SD. ^∗^*p* < 0.05, ^∗∗^*p* < 0.01. (c, d) REG*γ* WT or KO MEF cells were pretreated with GSK-3*β* inhibitor (CHIR99021, 10 *μ*M) for 12 hours, then 50 *μ*M of H_2_O_2_ was added for 30 minutes stimulation. After replacing H_2_O_2_ with fresh medium for 4 hours, cells were harvested for analyses of *Ho1* and *Nqo1* transcription by real-time qPCR. Statistical analyses were performed using GraphPad Prism and statistical significance was assessed by Student's *t*-test. Experiments were independently repeated six times. The data presented as mean values ± SD. ^∗^*p* < 0.05, ^∗∗^*p* < 0.01, ^∗∗∗^*p* < 0.001, ns: not significant. (e) REG*γ* WT or KO MEF cells were pretreated with GSK-3*β* inhibitor (CHIR99021, 10 *μ*M) for 12 hours, 50 *μ*M of H_2_O_2_ was added for 30 minutes stimulation. After replacing H_2_O_2_ with fresh medium for 12 hours, cells were harvested for analyses of Ho1 and Nqo1 protein level by western blot using indicated antibodies. (f) Experiments were independently repeated six times to do the quantitative and statistical analysis. The data presented as mean values ± SD. ^∗^*p* < 0.05, ns: not significant.

Inhibition of GSK-3*β* was reported to lead to Nrf2 nuclear accumulation and activation. Additionally, our previous study discovered that REG*γ* boosts GSK-3*β* decay in an ATP- and ubiquitin-independent manner [39]. Therefore, we hypothesized that REG*γ* promotes the nuclear retention and activation of Nrf2 through degrading GSK-3*β*. Faster degradation of GSK-3*β* in REG*γ*^+/+^ than REG*γ*^−/−^ cells was corroborated first (Figures 4(a) and 4(b)). Then, WT and REG*γ* deficient MEF cells were pretreated with 10 *μ*M of GSK-3*β* inhibitor (CHIR99021) for 12 hrs followed by incubation with 50 *μ*M of H_2_O_2_ for 30 minutes. *Ho1* and *Nqo1* mRNA level were examined by real-time PCR and gel-based PCR after removing H_2_O_2_ for 4 hours (Figures 4(c) and 4(d) and S2c). Western blot was employed to detect the protein level of Ho1 and Nqo1 after removing H_2_O_2_ for 12 hours (Figures 4(e) and 4(f)). The results displayed that H_2_O_2_ alone led in remarkable increase of Ho1 and Nqo1 mRNA and protein level in REG*γ* containing cells rather than in REG*γ* lacking cells (Figures 4(c)–4(f)). When pretreating cells with Gsk-3*β* inhibitor, less difference was observed between REG*γ*^+/+^ and REG*γ*^−/−^ group when compared with H_2_O_2_ alone treatment group (Figures 4(c)–4(f)). These studies illustrate that REG*γ* augments oxidative stress-induced activity of Nrf2 and expression of Ho1 and Nqo1 is at least partially mediated by a mechanism involving GSK-3*β* inhibition.

### 3.5. The Expression of REG*γ* Is Strengthened by Oxidative Stress

**Figure 5:**
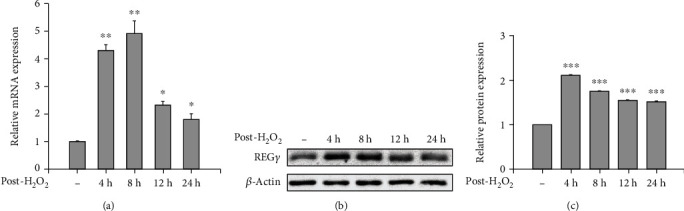
REG*γ* expression was upregulated by oxidative stress. (a) 293 cells were stimulated without (-) or with 50 *μ*M H_2_O_2_ for 2 hours, then H_2_O_2_ was replaced by fresh medium, and the cells were allowed to adapt for indicated time followed by qPCR. Experiments were independently repeated for 3 times and the data was presented as mean ± SD. ^∗^*p* < 0.05, ^∗∗^*p* < 0.01. (b) Cells were treated as in (a), and then the expression of REG*γ*, and *β*-actin was measured by western blot analysis. (c) The relative ratios of REG*γ* were represented by densitometric analysis. The results are representative of three independent experiments and expressed as means ± SD. ^∗∗∗^*p* < 0.001.

To facilitate the removal of oxidatively damaged proteins, ubiquitin-/ATP-independent degradation by the 20S is prominently enhanced because of the increased amount of free 20S in cells, which is the result of oxidative stress triggered disassembly of the 26S proteasome [52]. Since the REG*γ* proteasome is part of the ubiquitin-/ATP-independent degradation system, we explored the expression of REG*γ* upon oxidative stress. Intriguingly, quantitative RT-PCR (qRT-PCR) revealed that REG*γ* mRNA level was obviously upregulated after short period exposure of H_2_O_2_ in a time-dependent manner up to 8 hours, and then decreased steadily thereafter (Figure 5(a)). Notably, even 24 hours after oxidative stress, the REG*γ* protein still kept at a markedly high level (Figures 5(b) and 5(c)). These results point out that REG*γ* may be upregulated and maintained to provide a long-lasting protection for oxidative stress challenged cells.

### 3.6. H_2_O_2_ Induced REG*γ* Expression via Nrf2

**Figure 6:**
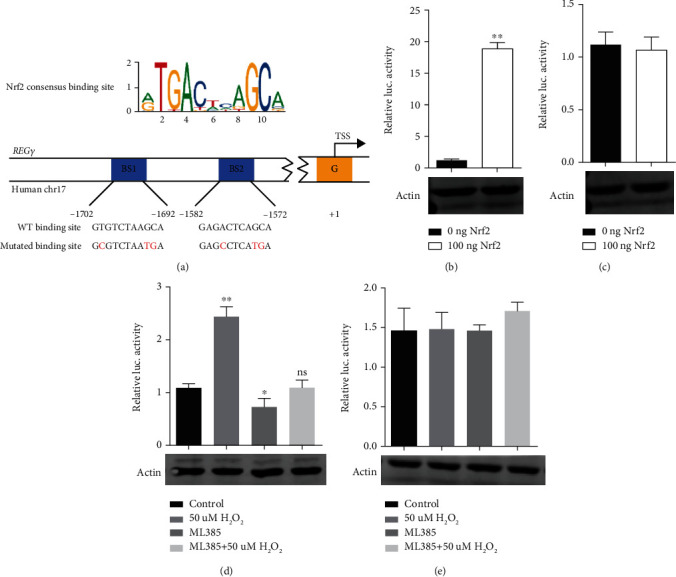
H_2_O_2_ upregulated REG*γ* via Nrf2. (a) Schematic diagram for Nrf2 consensus binding site (upper) and the luciferase reporter gene including wild type or mutated Nrf2 consensus binding sites (lower). 293 T cells were transiently transfected with 50 ng wild-typed (b) or mutated (c) REG*γ* luciferase reporter gene and 100 ng of Nrf2 plasmid. After 36 hours, the cells were collected for luciferase activity analysis. 293 T cells were transiently transfected with 50 ng wild-typed (d) or mutated (e) REG*γ* luciferase reporter gene and then treated with 10 *μ*M Nrf2 inhibitor (ML385) for 12 hours and 50 *μ*M H_2_O_2_ for 4 hours, respectively. Then, cells were harvested to determine the luciferase activity. *β*-Actin was used as the internal control. All experiments were independently repeated at least three times. Error bars represents ± SD. ^∗^*p* < 0.05; ^∗∗^*p* < 0.01, ns: not significant.

In order to test whether REG*γ* would be transcriptionally modulated by Nrf2, first we transiently transfected HaCaT cells with Nrf2 plasmid or Nrf2 specific siRNAs. Then, the mRNA level of REG*γ* was inspected by gel-based PCR. It turned up that REG*γ* was upregulated by overexpression of Nrf2 (Figure S3a) and downregulated by knocking down of Nrf2 (Figure S3b). Next, we analyzed the promoter of human *REGγ* genes for binding sequences of Nrf2. We focused on the regions from 1.7 kb upstream to 1 kb downstream of the transcription start site. Using the JASPAR database, two potential Nrf2 consensus binding sites were identified. Then, we generated luciferase reporter gene including wild type or mutated Nrf2 consensus binding sites (Figure 6(a)). Transient transfection of Nrf2 dramatically increased the activity of wild-typed luciferase reporter gene (Figure 6(b)). After we introduced site-specific mutagenesis in the potential binding site, the REG*γ* luciferase reporter gene became unresponsive (Figure 6(c)). Alternatively, specific Nrf2 inhibitor (ML385) was employed to blunt the activation of endogenous Nrf2. It emerged that H_2_O_2_ substantially stimulated wild-typed reporter gene activation instead of the mutant one (Figures 6(d) and 6(e)). Meanwhile, Nrf2 inhibitor evidently suppressed the reporter response to H_2_O_2_ treatment (Figure 6(d)). To sum up, these findings establish a positive feedback regulation loop between REG*γ* and Nrf2 to protect cells from oxidative stress driven cell senescence.

## 4. Discussion

**Figure 7:**
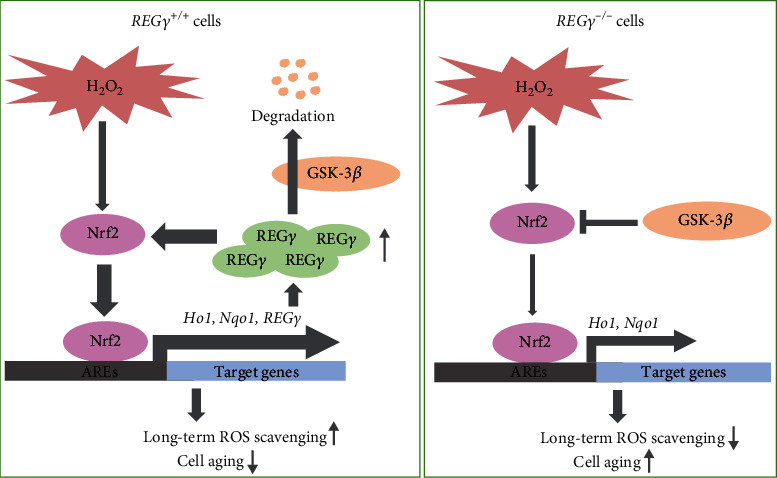
Proposed working model. Upon H_2_O_2_ stimulation, Nrf2 is activated and translocated into nucleus to upregulate antioxidant enzyme Ho1 and Nqo1, as well as REG*γ*. After removal of H_2_O_2_, REG*γ* augments and extends the activity of Nrf2 by directing GSK-3*β* degradation, which finally leads to long-term ROS scavenging and cell aging protection.

A powerful antioxidant system has evolved to maintain redox homeostasis of aerobic creatures (including humans) to prevent disease and aging [53, 54]. REG*γ*, as a member of the 11S proteasome activator family, degrades a variety of substrate proteins and participates in many important physiological and pathological processes [34]. In present study, we shed light on a REG*γ*-mediated unknown mechanism responsible for regulation of antioxidative ability and cell aging under oxidative stress and proposed the following working model. In REG*γ* normal cells, H_2_O_2_ stimulation led to immediate Nrf2 activation and subsequent elevation of downstream target genes to clear out the oxygenic free radicle. Concurrently, REG*γ*, as a new identified target gene of Nrf2, was upregulated and maintained long period activity of Nrf2 via directing degradation of GSK-3*β*, contributing to long-term ROS removal and cell aging delay. However, in REG*γ* deficient cells, the negative regulation of Nrf2 by GSK-3*β* could not be antagonized, which resulted in increased ROS accumulation and cell aging (Figure 7). In conclusion, our research delineated a new regulatory layer of Nrf2 by REG*γ* and a positive feedback loop between Nrf2 and REG*γ* in promoting long-term ROS scavenging, hence delaying oxidative stress-induced cell aging.

Free radicals such as ROS can produce cumulative oxidative damage to macromolecules and lead to cellular senescence [55]. The 20S proteasome appears to be the main player in the process that recognizes and degrades oxidatively damaged proteins [56]. At first, redox modifications quickly boost the catalytic activity and proteolytic capacity of preexisting 20S proteasomes. Furthermore, short-term oxidative stress causes the 26S proteasome complex to disassemble into 20S and 19S particles [57]. Previously, our laboratory has depicted that the association of REG*γ* with 20S proteasome was enhanced by acute oxidative stress to promote proteasome-dependent proteolysis of oxidized protein substrates [47]. Our present study further announced that REG*γ* could act as antioxidant to resist oxidative stress and slow down cell aging in the adaptation stage after oxidative stress. It effectively maintained ROS concentration at a low level and provided long-lasting antioxidant effect, highlighting the link between ubiquitin-independent proteasome complex and the long-term adaption of cells to oxidative stress.

Previous study demonstrated that REG*γ* knockout or knockdown significantly elevates SOD2 expression in mouse heart tissues or in AC16 cell lines [58]. In accordance with this, we also observed that SOD2 level was higher in REG*γ* deficient MEF cells than in WT MEF cells in unstressed normal condition, however had no effect on the expression of SOD1 (data not shown), which may explain why ROS in REG*γ* knockout cells was temporarily less than in REG*γ* normal cells after exposure to H_2_O_2_ (Figure 2 and S1). We prefer that this transiently lower ROS level in REG*γ* KO cells is due to a rescue or a compensation effect mediated by other factors, such as the SOD2, rather than the direct function of REG*γ* to regulate the ROS level in this particular REG*γ* lacking situation. Since a small amount of ROS act as signaling molecules and has health-promoting effects in the cells, REG*γ* deficient cells may be unable to produce enough ROS and fail to completely elicit and elongate the antioxidant response, as shown in Figure 3 in current data. After allowing the cells to recover for a while, REG*γ* lacking cells had considerably more ROS accumulation due to loss of prolonged transcriptional activity of Nrf2 by REG*γ*. These studies depict complicated regulation of oxidative stress response by REG*γ*. How cells sense the ROS level to switch REG*γ*-20S proteasome for the off/on regulation of Nrf2 activity under oxidative stress or normal conditions deserves further studies.

It is well accepted that the equilibrium between antioxidant and oxidant is disrupted during aging [59]. However, the change of Nrf2 activity with age may depend on species, tissues, and cell types [9]. REG*γ*-deficient mice have been reported to develop premature aging phenotypes [46]. Here, our study evidenced that the deactivation of Nrf2 mediated by GSK-3*β* under prolonged exposure to oxidative stress was predominantly rescued by REG*γ*. Combined with our recent observation that REG*γ* level was declined with age (data not shown), we suggest that there is a bias towards deregulation of REG*γ* during aging, and a decay of REG*γ* can further accelerate aging process. Whether this regulation exists in divergent cell types, tissues or species deserve more systematic studies.

## 5. Conclusions

Overall, our findings demonstrate a scenario where REG*γ*, the 11S family of proteasome activator, prolongs the nuclear retention of Nrf2 and the expression of its downstream antioxidant enzymes Ho1 and Nqo1, to reduce the ROS accumulation, enhance the oxidant resistance, and eventually protect against oxidative stress-mediated cell senescence. These results make REG*γ* a promising drug target since pharmacological manipulation of REG*γ* could have potential benefits on oxidative damage-related disorders and aging.

## Data Availability

The supporting information can be downloaded at: http://www.xxx.xxx/xxx/. Figure S1: the level of ROS in REG*γ* WT and KO cells after short time exposure of H_2_O_2_. Figure S2: Nrf2 was not a substrate of REG*γ*. Figure S3: up- and downregulation of REG*γ* by overexpression and knockdown of Nrf2.

## Abbreviations

Nrf2: Nuclear factor erythroid 2-related factor 2
H_2_O_2_: Hydrogen peroxide
ROS: Reactive oxygen species
SOD1: Superoxide dismutase 1
SOD2: Superoxide dismutase 2
Ho1: Heme oxygenese-1
Nqo1: NAD(P)H quinone acceptor oxidoreductase 1
WT: Wild type
KO: Knockout
MEF: Mouse embryonic fibroblast
SA *β*-Gal: Senescence-associated *β*-galactosidase
GSK-3*β*: Glycogen synthase kinase-3*β*
SD: Standard deviation.

## Supplementary Materials

Supplementary Materials Figure S1: the level of ROS in REG*γ* WT and KO cells after short time exposure of H_2_O_2_. Figure S2: Nrf2 was not a substrate of REG*γ*. Figure S3: up- and downregulation of REG*γ* by overexpression and knockdown of Nrf2.
