# Supplementary material for: Reciprocal REGγ-Nrf2 Regulation Promotes Long Period ROS Scavenging in Oxidative Stress-Induced Cell Aging
Source: Oxid Med Cell Longev. 2023 Jan 10;2023:4743885. doi: 10.1155/2023/4743885 (PMC9845040; doi:10.1155/2023/4743885)
Supplement: Supplementary Materials — Figure S1: the level of ROS in REGγ WT and KO cells after short time exposure of H2O2. Figure S2: Nrf2 was not a substrate of REGγ. Figure S3: up- and downregulation of REGγ by overexpression and knockdown of Nrf2. [file 4743885.f1.docx]

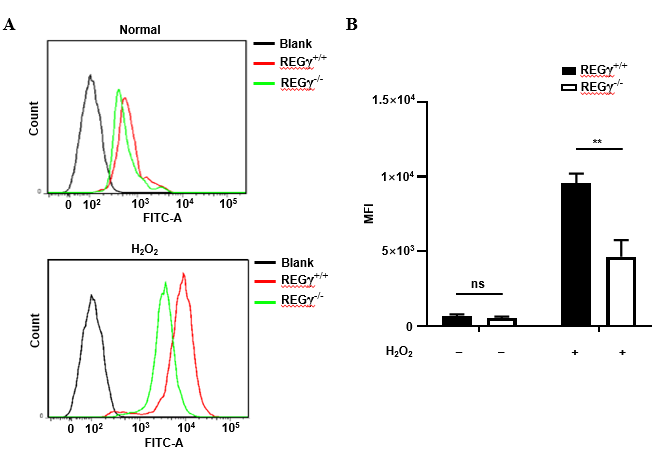


**Fig S1. The level of ROS in REGγ WT and KO cells after short time exposure of H_2_O_2_.** (**A**) 293 WT and KO cells were stimulated with 50 μM H_2_O_2_ for 30 min, then DCFH-DA ROS fluorescence probe was added and incubated for 20 min. Mean fluorescence intensity (MFI) was measured by flow cytometry. (**B**) The MFI values of each group were statistically analyzed (n=3). ∗∗*p* < 0.01, ns = not significant.


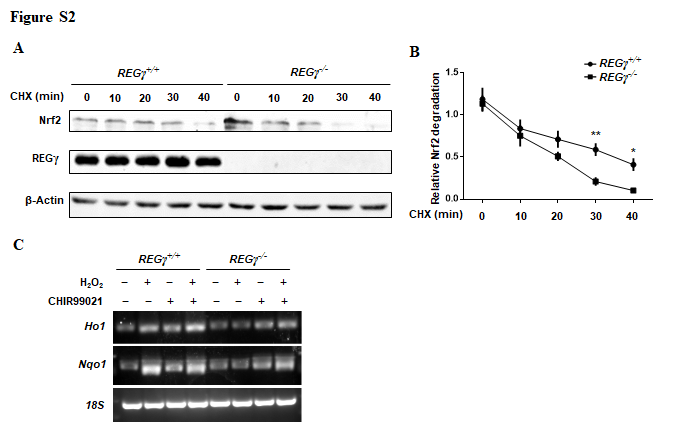


**Fig S2. Nrf2 was not a substrate of REGγ.** (**A**) 100 μg/mL cycloheximide was added to REGγ WT or KO MEF cells, then the cells were harvested at indicated time. The stability of Nrf2 was detected by Western blot. (**B**) Experiments in (A) were repeated for four times and Nrf2 degradation was analyzed quantitively by Image J software. The data presented as mean values ± SD. *p<0.05, **p<0.001. **(C)**. Total RNA was prepared from WT or KO MEF cells treated as in Figure 4c**.** Gel based PCR was performed using the primers as indicated under “Materials and Methods”. The experiments were repeated for three times.


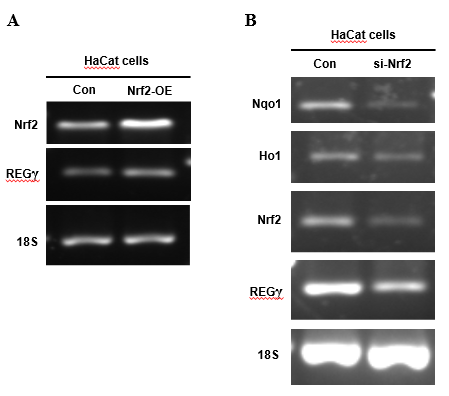


**Fig S3.** **Up- and down-regulation of REGγ by overexpression and knockdown of Nrf2.** (A) Hacat cells were transfected with 500ng of control or Nrf2 plasmid and then collected for REGγ mRNA level detection by RT-PCR. (**B**) Hacat cells were transfected with 50nM negative control siRNA or Nfr2 specific siRNA for 48 hours and then were collected for indicated mRNA level detection by RT-PCR. All the experiments were repeated for three times.
